# Risk Evaluation and Molecular Characterisation of *AtNPR1* Transgenic Citrus Lines Tolerant to Citrus Greening Disease

**DOI:** 10.1111/pbi.70394

**Published:** 2025-10-13

**Authors:** Paula Rios Glusberger, Benjamin Merritt, Cheng Liu, Yu Wang, Janice Zale, Hao Wu, Michel Canton, Michael Braverman, Eric W. Triplett, Zhonglin Mou

**Affiliations:** ^1^ Microbiology and Cell Science Department Institute of Food and Agricultural Sciences, University of Florida Gainesville Florida USA; ^2^ Citrus Research and Education Center, Food Science and Human Nutrition Department Institute of Food and Agricultural Sciences, University of Florida Lake Alfred Florida USA; ^3^ Citrus Research and Education Center, Horticulture Department Institute of Food and Agricultural Sciences, University of Florida Lake Alfred Florida USA; ^4^ The IR‐4 Project North Carolina State University Raleigh North Carolina USA

**Keywords:** *AtNPR1*, citrus biotechnology, citrus greening, citrus transformation, disease resistance, genetically modified organisms, HLB tolerance, sustainable agriculture

## Abstract

Citrus greening disease, or Huanglongbing (HLB), has caused devastating losses to citrus production in Florida, with yields declining by over 90% since 2005. Despite extensive efforts, no sustainable solution has been widely effective. Here, transgenic ‘Hamlin’ sweet orange lines engineered to constitutively express the *Arabidopsis NPR1* (*AtNPR1*) gene, a key regulator of systemic acquired resistance, are evaluated for health and environmental risks. These citrus lines exhibit strong HLB tolerance, with reduced disease symptoms, sustained fruit production, and no apparent negative phenotypic abnormalities. Comprehensive risk assessment reveals minimal exposure, health, or environmental risk. The AtNPR1 protein is: (1) barely detectable in fruit, (2) rapidly degraded in simulated gastrointestinal fluids, and (3) not similar to known allergens or toxins. Whole‐genome sequencing identified the T‐DNA insertion sites as heterozygous in either chromosome 1 or 6, with no disruptions in known fruit‐producing genes. PCR markers were developed for rapid line identification. The selected lines are currently in a small field trial under high HLB pressure and continue to exhibit low visual HLB symptoms and positive horticultural traits. These findings support the initial requirements for regulatory approval of these transgenic citrus varieties, offering a promising strategy for sustainable citrus production.

## 1 Introduction

Citrus greening disease, also known as Huanglongbing (HLB), was first observed in Florida citrus in 2005 (Bové 2006). Within a few years, it spread to every citrus‐growing region of the state, devastating the Florida citrus industry and now posing a significant threat to citrus production in California and Texas (Graham et al. 2020). The latest Florida Citrus Statistics 2022–2023 report a dramatic decline in citrus production, from 13 million tons in 2005 to merely 800 000 tons by the end of the 2023 season (USDA and NASS 2024), a nearly 94% drop largely due to HLB. The disease is caused by a phloem‐limited, uncultured alphaproteobacterium, *Candidatus* Liberibacter asiaticus (*C*Las), vectored into the citrus phloem by the Asian citrus psyllid, *Diaphorina citri* (Garnier et al. 1984; Jagoueix et al. 1994). Symptoms of HLB include leaf mottling, stunted growth, yellowing foliage, twig dieback, and small, misshapen fruit that remain dull green (Garnier and Bové 1993; Gottwald et al. 2007). Despite many efforts by scientists worldwide, cost‐effective and environmentally sustainable management practices for controlling HLB have not been successful (Li et al. 2020). The USDA and University of Florida citrus breeding programs have made progress in producing HLB‐tolerant citrus varieties through traditional breeding (Stover et al. 2016; Zapien‐Macias et al. 2022). However, achieving commercially acceptable oranges and orange juice has proven difficult due to the lengthy juvenile stage of citrus trees and the lack of naturally resistant germplasm (Stover et al. 2019, 2020). Biotechnological methods, including genetic engineering of HLB‐resistant or ‐tolerant citrus varieties, offer a solution to the limitations of conventional breeding (Kumar et al. 2023). One approach is the use of transgenic citrus lines expressing the *Arabidopsis thaliana NONEXPRESSOR OF PATHOGENESIS‐RELATED GENES1* (*AtNPR1*) gene (Dutt et al. 2015; Robertson et al. 2018; Zhang et al. 2010).

NPR1 is a key positive regulator of systemic acquired resistance (SAR), which is activated by salicylic acid and drive the expression of *PATHOGENESIS‐RELATED* (*PR*) genes to provide broad‐spectrum, long‐lasting protection against pathogens (Delaney 1997; Fu and Dong 2013). The NPR1 protein interacts with many transcription and chromatin‐related proteins, revealing complex mechanisms in plant disease resistance (Backer et al. 2019; Powers et al. 2024). Furthermore, crystal structures have elucidated the complex assembly of the NPR1 protein with TGA transcription factors to regulate the defence transcriptome (Kumar et al. 2022). In *Arabidopsis*, mutants defective in *NPR1* lose their ability to induce SAR and show increased susceptibility to pathogens (Cao et al. 1994; Delaney et al. 1995). In contrast, transgenic *Arabidopsis* overexpressing *AtNPR1* show elevated resistance to pathogens with no obvious detrimental effects to the plant (Cao et al. 1998). The *AtNPR1* gene has been introduced into multiple crops, including rice, wheat, soybean, grape, carrot, tomato, citrus, and strawberry (Silva et al. 2018). Its overexpression effectively enhances disease resistance, although in a few cases, detrimental effects have been observed (Silva et al. 2018).

In citrus, overexpression of *AtNPR1* increased resistance to citrus canker in both ‘Duncan’ grapefruit and ‘Hamlin’ orange, directly correlating with *AtNPR1* expression levels (Zhang et al. 2010). In ‘Hamlin’ orange, resistance to *Xanthomonas citri* subsp. citri, the pathogen causing citrus canker, was linked to higher expression of *EDS1* and *PR2* genes post‐inoculation (Boscariol‐Camargo et al. 2016). In greenhouse and field studies, reduced HLB severity occurred in ‘Hamlin’ and ‘Valencia’ oranges overexpressing *AtNPR1*, associated with increased *PR1* and *PR2* expression (Dutt et al. 2015). Molecular studies show that *AtNPR1* in transgenic citrus interacts with the citrus innate defence pathway, demonstrated by increased transcriptional changes and protein–protein interactions (Qiu et al. 2020). Studies on the mechanisms of HLB tolerance revealed that *AtNPR1* overexpression suppresses pathogen‐induced overaccumulation of callose and reactive oxygen species (ROS) (Sarkar et al. 2024). Callose plugging and ROS overaccumulation have been shown to be responsible for HLB symptoms and cell death (Achor et al. 2010, 2020; Koh et al. 2012; Ma et al. 2021). In prolonged greenhouse studies, the ‘Duncan’ grapefruit and ‘Hamlin’ transgenic lines expressing *AtNPR1* have consistently exhibited robust HLB tolerance for over nine years, with second‐ and third‐generation progeny maintaining similar levels of tolerance under severe disease pressure (Robertson et al. 2018).

While public opinion in the United States remains divided on the use of genetically modified (GM) crops as of 2020, there is a gradual acceptance and growing recognition that GM crops can address climate change, food security, and sustainability issues (Kennedy and Thigpen 2020; Verma et al. 2021). Also, the recent success of widely available transgenic crops, such as Arctic Apple (Stowe and Dhingra 2021), in the United States market indicate shifts in public opinion (ISAAA 2015). *Agrobacterium*‐mediated transformation is the most common method used in citrus, and more efficient methods are being explored (Conti et al. 2021; Poles et al. 2020). For example, virus‐mediated transformation is currently being studied to expedite gene editing in plants (Belaffif et al. 2025).

In this work, we selected five 'Hamlin' lines with strong HLB tolerance—these lines were previously transplanted in 2019 to a field site in Ft. Pierce, Florida. Two of the lines—13–3 and 13–29—studied in Robertson et al. (2018), and lines 24–25, 26–36 and 35–30 were generated later via mature tissue transformation to expedite fruiting (Canton et al. 2024). Lines transformed with mature tissue are currently producing oranges in the field, and the others are beginning fruit production as of Spring 2025. Protein assay data indicate that health risks and exposure to the AtNPR1 protein are minimal to none for human consumption and the environment. The T‐DNA insertion in the genome of each line was identified, and the transgenes do not appear to impact fruit‐producing genes. PCR‐based markers were generated to identify and track each line. These data are being used in our applications to regulatory agencies in the United States.

## 2 Results

### 2.1 AtNPR1 Protein Is Minimally Detected in Fruit Tissues, and Is Rapidly Degraded by Proteases In Vitro, With no Homology to Known Allergens and Toxins

**FIGURE 1:**
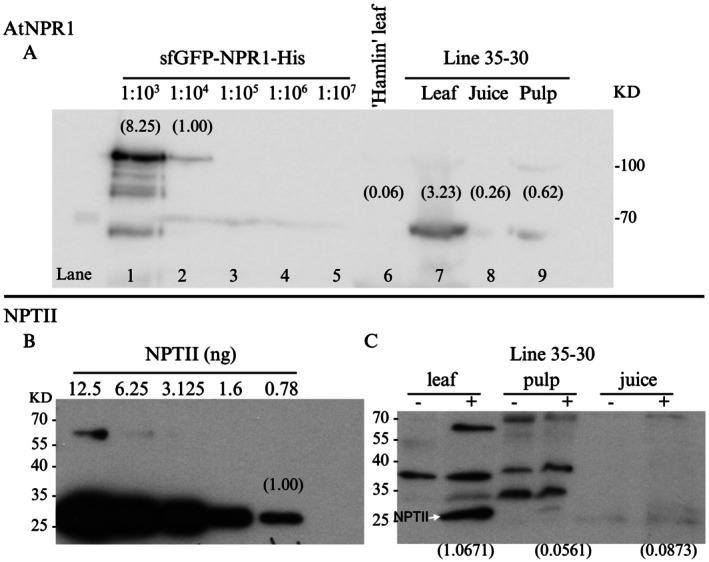
Western blot analysis of AtNPR1 and NPTII protein levels in transgenic citrus tissues. (A) A standard curve was generated using serial dilutions of purified sfGFP‐NPR1‐His fusion protein (lanes 1–5), with band intensities ranging from high to low concentrations. The band from the 1:10^4^ dilution (lane 2) was normalised to 1.00 and used as the reference for quantifying AtNPR1 levels in 'Hamlin' wild‐type leaf (lane 6) and transgenic line 35–30 tissues—leaf, juice and pulp (lanes 7–9). Relative protein levels, calculated using ImageJ by comparing band intensities, are shown in parentheses above each band. (B) A standard curve for NPTII protein was established using known quantities (12.5–0.78 ng), with the 12.5 ng band normalised to 1.00. (C) Western blot analysis of NPTII expression in transgenic line 35–30 tissues—leaf, pulp and juice—under treated (+) and untreated (−) conditions. Band intensities were quantified and normalised against the NPTII standard curve. Normalised values are shown in parentheses. The white arrow indicates the NPTII band.

**FIGURE 2:**
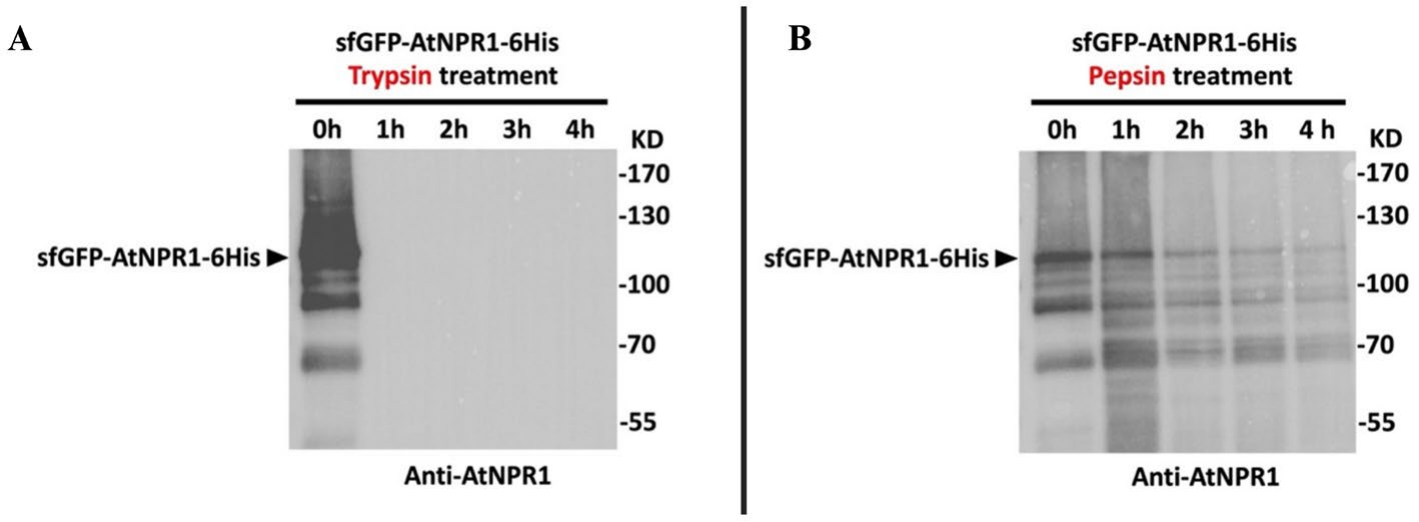
Proteolytic digestion of AtNPR1 protein by trypsin and pepsin. In vitro digestion assays were conducted to assess the stability of sfGFP‐AtNPR1‐6His against proteolytic enzymes. Purified sfGFP‐AtNPR1‐6His (1.67 μg) was treated with 0.085 μg of either trypsin (A) or pepsin (B), maintaining an enzyme‐to‐substrate ratio of 1:20 (w/w), for 0, 1, 2, 3 and 4 h. Samples were analysed by Western blot using an anti‐AtNPR1 antibody. Complete degradation of the full‐length protein by trypsin was observed within 1 h (A), while pepsin treatment resulted in partial degradation and multiple smaller fragments over the 4‐h time course (B).

Among the three lines generated via mature tissue transformation, line 35–30 accumulates the highest level of the AtNPR1 protein. AtNPR1 was detected in the leaf tissue of line 35–30 at an approximate concentration of 2.91 μg/g of fresh weight, 4.68 μg/L in fruit juice and 0.19 μg/g in pulp (Figure 1A). The neomycin phosphotransferase II (NPTII) protein was detected at an approximate concentration of 0.055 μg/g in leaf tissue, 0.091 μg/L in fruit juice, and 0.001 μg/g in pulp (Figure 1B,C). Proteolytic digestion assays demonstrated that AtNPR1 was fully degraded by trypsin within one hour and gradually degraded over four hours in the presence of pepsin (Figure 2A,B), simulating gastrointestinal conditions. Bioinformatic risk assessments using AllergenOnline (Goodman et al. 2016) and Allermatch (Fiers et al. 2004), revealed no homology between AtNPR1 and known allergens, based on the recommended threshold of ≥ 35% identity over 80 amino acids (Figures S1 and S2). Similarly, toxicity screening tools (CSM‐Toxin and ToxDL) found no matches to characterised toxic proteins (Figure S3).

Together, these results suggest that AtNPR1 poses negligible risk as a food additive. Its minimal presence in fruit juice and pulp, rapid in vitro degradation by digestive enzymes, and lack of similarity to known allergens or toxins supports its safety profile.

### 2.2 Nutritional Composition of Transgenic Orange Juice Is Comparable to Non‐Transgenic Controls

Orange juice from transgenic lines 35–30 and 24–25 showed similar overall nutrient profiles to the non‐transgenic 'Hamlin' control. Slight increases were observed in calorie content (35.4 kcal/100 mL in 35–30 vs. 29.1 kcal/100 mL in the control) and total sugars (6.83 g/100 mL in 35–30 vs. 5.64 g/100 mL). Line 24–25 had the greatest protein content (0.97 g/100 mL), while the control had slightly higher calcium (10.5 mg/100 mL vs. 6.3–6.9 mg in transgenic lines). Other nutrients, including fats, sodium, cholesterol, and vitamin D, remained below detection limits across all samples. These results indicate no substantial nutritional differences between transgenic and non‐transgenic orange juice (Table 1).

**TABLE 1:** Nutrient composition of juice from transgenic and non‐transgenic citrus lines.

Nutrient	Unit	35–30		24–25		Hamlin control	
		Result per 100 g	Result per 100 mL	Result per 100 g	Result per 100 mL	Result per 100 g	Result per 100 mL
Calories	kcal	34.4	35.4	32.6	33.7	28.4	29.1
Total fat	g	0.11	0.11	< 0.10	< 0.10	< 0.10	< 0.10
Monounsaturated fat	g	< 0.10	< 0.10	< 0.10	< 0.10	< 0.10	< 0.10
Polyunsaturated fat	g	< 0.10	< 0.10	< 0.10	< 0.10	< 0.10	< 0.10
Saturated fat	g	< 0.10	< 0.10	< 0.10	< 0.10	< 0.10	< 0.10
Trans fat	g	< 0.10	< 0.10	< 0.10	< 0.10	< 0.10	< 0.10
Cholesterol	mg	< 0.80	< 0.80	< 0.80	< 0.80	< 0.80	< 0.80
Sodium	mg	< 0.2	< 0.21	< 0.2	< 0.21	< 0.2	< 0.20
Potassium	mg	144	148	181	187	161	165
Total carbohydrate	g	7.60	7.80	7.20	7.40	6.30	6.50
Dietary fibre	g	< 0.47	< 0.48	< 0.47	< 0.49	< 0.47	< 0.48
Sugars	g	6.63	6.83	6.14	6.35	5.5	5.64
Fructose	g	2.00	2.06	1.80	1.86	1.50	1.54
Glucose	g	1.80	1.85	1.80	1.86	1.40	1.4
Lactose	g	< 0.25	< 0.26	< 0.25	< 0.26	< 0.25	< 0.26
Maltose	g	< 0.25	< 0.26	< 0.25	< 0.26	< 0.25	< 0.26
Sucrose	g	2.84	2.92	2.54	2.63	2.65	2.72
Protein (*F* = 6.25)	g	0.75	0.77	0.94	0.97	0.79	0.81
Calcium	mg	6.10	6.30	6.70	6.90	10.2	10.5
Iron	mg	< 0.10	< 0.10	< 0.10	< 0.10	< 0.10	< 0.10
Moisture	g	91.2	94.0	91.5	94.6	92.5	94.8
Ash	g	0.30	0.31	0.27	0.28	0.34	0.35
Vitamin D2	mcg	< 0.75	< 0.77	< 0.75	< 0.78	< 0.75	< 0.77
Vitamin D3	mcg	< 0.55	< 0.57	< 0.55	< 0.57	< 0.55	< 0.56
Total vitamin D	mcg	< 0.55	< 0.57	< 0.55	< 0.57	< 0.55	< 0.56

### 2.3 AtNPR1 Protein Shares High Sequence Similarity With NPR1 Homologues in Edible *Brassica* Species

**FIGURE 3:**
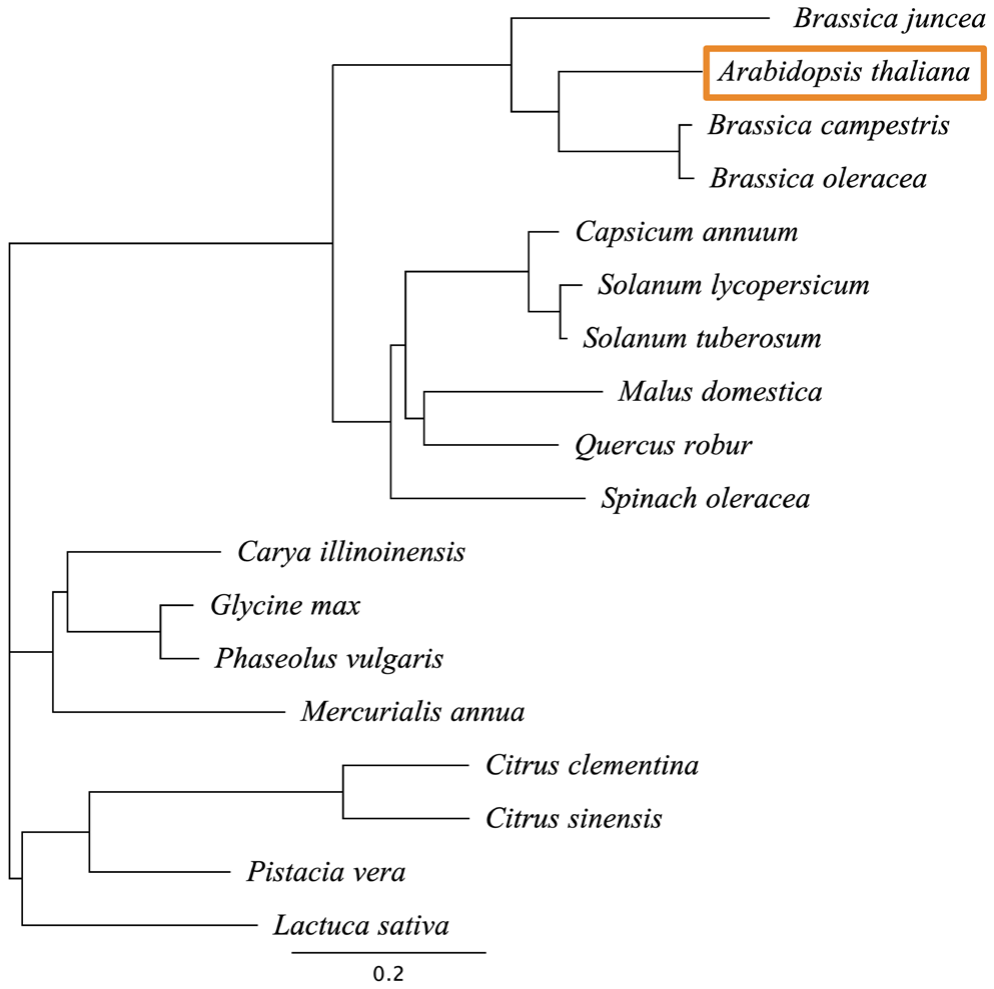
Neighbour‐Joining phylogenetic tree of NPR1 proteins from diverse plant species. A Neighbour‐Joining tree was constructed based on pairwise alignments of all sequence pairs of NPR1 protein from 18 plant species, using Geneious Prime 2025.1.2. The tree includes *A. thaliana* marked with an orange box and several of its edible relatives in the Brassicaceae family, as well as other agriculturally relevant species. Seventeen of the species represented are edible, including all *Brassica* species and *A. thaliana* itself. Scale bar represents evolutionary distance.

A Neighbour‐Joining phylogenetic tree of NPR1 proteins from various plant species, including edible members of the Brassicaceae family, shows that highly similar homologues of *A. thaliana* NPR1 are present in commonly consumed crops. Notably, *Brassica campestris* (field mustard) and *Brassica oleracea* (wild cabbage)—ancestors of many cruciferous vegetables—share 72% identity and 86.5% similarity at the amino acid level with *A. thaliana* NPR1 (Figure 3 and Table S1).

### 2.4 Transgenic Lines Continue to Display HLB Tolerance Despite Continued *C*Las Infection

**FIGURE 4:**
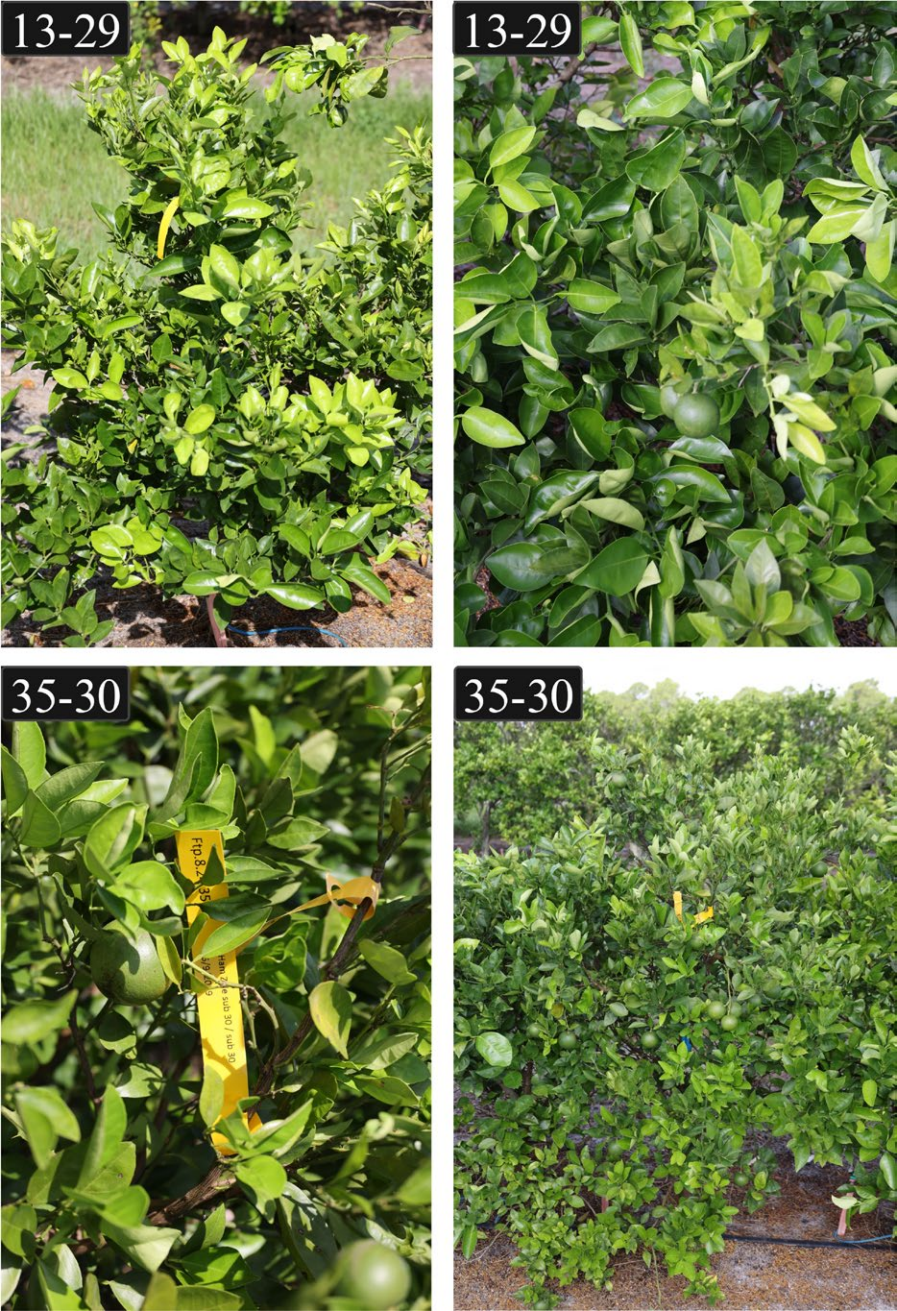
Field images of transgenic 'Hamlin' citrus lines 13–29 and 35–30 were taken in July 2024. The top two panels show line 13–29 and the bottom two panels show line 35–30. These trees are part of a field trial located in Ft. Pierce, Florida, a region with high HLB disease pressure.

The five selected 'Hamlin' transgenic lines under regulatory evaluation are currently maintained in a field site in Ft. Pierce, Florida, where HLB disease pressure is high. HLB tolerance in lines 13–3 and 13–29 was previously reported by Robertson et al. (2018), while lines 24–25, 26–36, and 35–30 were generated via mature tissue transformation to accelerate fruiting. All five lines continue to have little to no visible HLB symptoms and are considered tolerant (Figure 4; Figure S4). Despite their phenotypic tolerance, all lines remain *C*Las‐positive as determined by qPCR analysis (Figure S5).

### 2.5 T‐DNA Insertions Within Genes Have Not Affected Phenotype or Fruit Development to Date and Serve as Markers for Product Identification

Chromosome locations of the T‐DNA insertion sites were determined using the *Citrus sinensis* reference genome DVS_A1.0 to assess whether any known coding regions were potentially affected (Table 2; Figure S6). For line 13–3, the right border (RB) of the T‐DNA maps to chromosome 6, adjacent to *ZIP5* (zinc transporter 5) and *bHLH75* (transcription factor bHLH75). A *RPS7* gene (40S ribosomal protein S7) is present on both sides of the insertion site, but its coding sequence does not appear to be interrupted. In line 13–29, the insertion is located on chromosome 1, approximately 2 kb from *OPR3* (12‐oxophytodienoate reductase 3); an uncharacterised gene identified via NCBI BLAST lies further downstream, but no coding regions are directly intersected.

**TABLE 2:** T‐DNA insertion sites and nearby gene annotations in transgenic citrus lines.

Transgenic line	Predicted chromosome location	T‐DNA orientation relative to the chromosome	Gene ID	Gene/Ortholog name
13–3	6	Reverse	**LOC102609987**	**40S ribosomal protein S7 (at insertion site)**
			LOC102610913	Zinc transporter 5 (5′ adjacent)
			LOC102610603	Transcription factor bHLH75 (3′ adjacent)
13–29	1	Forward	LOC102631049	12‐oxophytodienoate reductase 3 (5′ adjacent)
			LOC107174913	Uncharacterised protein (5′ adjacent)
24–25	6	Reverse	None found	None found
26–36 and 35–30	1	Forward	**LOC102628729**	**Heme oxygenase 1 chloroplastic (at insertion site)**

*Note:* Bold gene ID and gene name have the T‐DNA inserted within the gene.

Lines 26–36 and 35–30 were found to be clones, with identical T‐DNA insertion sites located within the *HO1* gene (heme oxygenase 1, chloroplastic) on chromosome 1, as confirmed by Clustal Omega alignment showing 99.99% pairwise identity. No known or predicted genes were found at or near the insertion site in line 24–25.

The expression of the interrupted genes in lines 13–3, 26–36, and 35–30 was assessed using qPCR. While the expression of *RPS7* is not significantly affected by the T‐DNA insertion in line 13–3, the expression of *HO1* is reduced in both lines 26–36 and 35–30, though the reduction in line 35‐30 is not significant (Figure S7).

**FIGURE 5:**
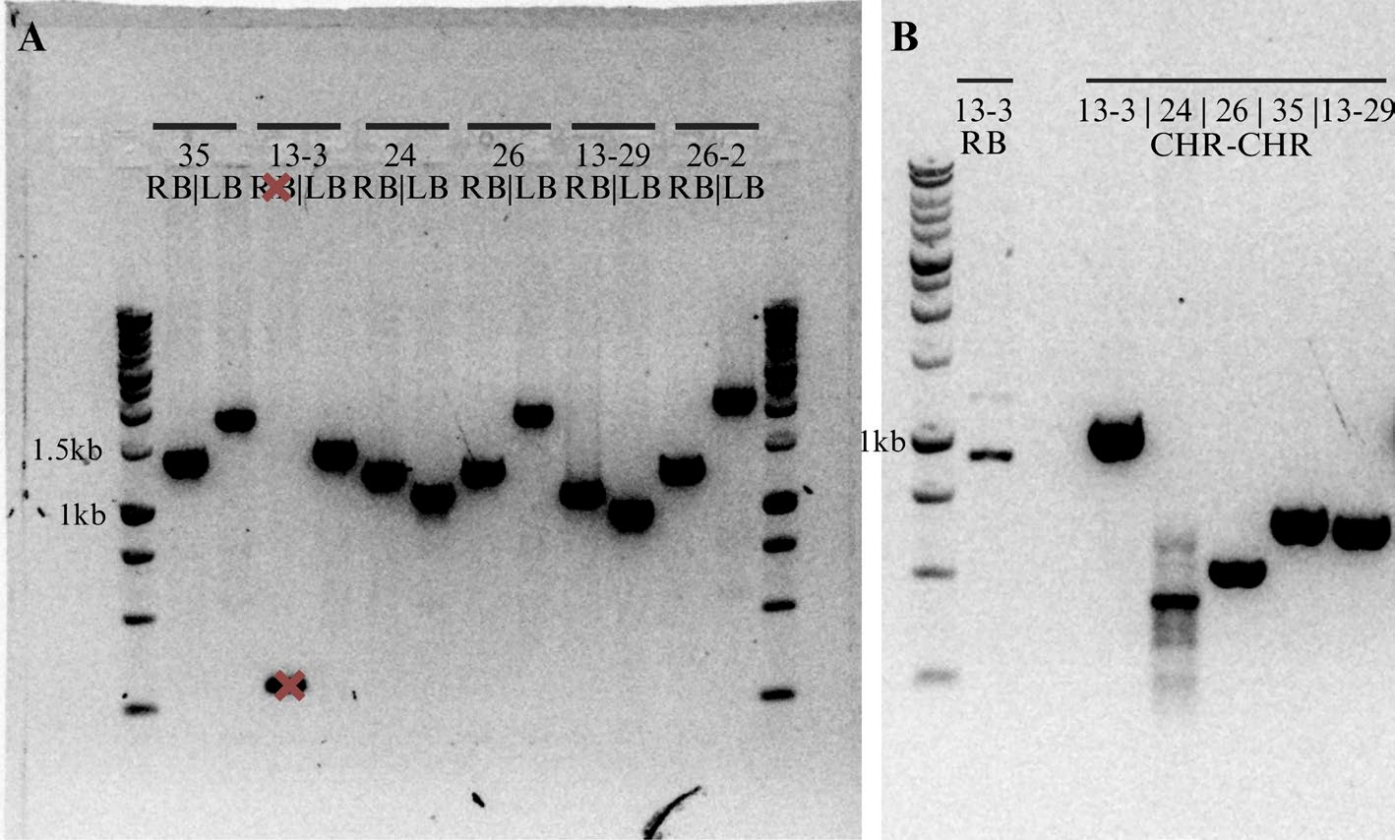
PCR confirmation of T‐DNA insertion sites and AtNPR1 heterozygosity. (A) Gel image showing PCR products amplified at the right border (RB) and left border (LB) of the T‐DNA insertion site for each transgenic line. Primers were designed to span ~500 bp of chromosome sequence and ~500 bp of T‐DNA to generate amplicons specific to the insertion site. The RB amplification for line 13–3 initially failed (marked with an ‘X’) and 26–2 represents a secondary primer set not used for line identification. (B) Improved RB primer design successfully amplified the insertion site for 13–3 (lane 2). Chromosome‐to‐chromosome (CHR–CHR) amplicons (lanes 4–8) confirm heterozygosity in all lines, as products are shorter than the full 6.5 kb T‐DNA.

These defined insertion sites also provided unique molecular markers for transgenic line identification. Primer sets were designed to amplify the RB and left border (LB) junctions, spanning ~500 bp of T‐DNA and chromosome sequence. Predicted amplicon sizes were confirmed by gel electrophoresis (Figure 5A), and Sanger sequencing of each product aligned to the insertion site contigs with 95.2%–99.8% identity (Table S2). All lines were confirmed to be heterozygous for the T‐DNA insertion, based on successful amplification of RB, LB and chromosome‐to‐chromosome junctions. As expected for heterozygous insertions, the CHR–CHR amplicons—spanning the intact chromosome in the absence of the T‐DNA—were shorter than the full 6.5 kb T‐DNA sequence (Figure 5B).

## 3 Discussion

Here, evidence is presented to support the notion that *AtNPR1* transgenic lines of citrus are ideally suited for commercial production, given their extremely low potential for health risks. According to the Environmental Protection Agency (EPA), health risk is a product of hazard and exposure (US EPA 1991), and in every area of concern, the AtNPR1 protein is not considered a hazard and the exposure is minimal—this is consistent with the EPA's risk assessment recommendations (EPA 2014).

Hazard assessments of allergenic and toxic potential—using tools recommended by the EPA—of the AtNPR1 protein sequence did not identify it as an allergen or toxin. In addition, the source of the transgene is *A. thaliana* (NCBI:txid3702) in the Brassicaceae family of plants from which edible cruciferous vegetables are derived (Petruzzello 2025). The AtNPR1 protein sequence shares 72% identity and 86.5% similarity with NPR1 homologous proteins from *B. campestris* and *B. oleracea* , based on pairwise alignments of all protein sequence pairs performed. This puts the AtNPR1 protein at a low probability of allergenicity according to the allergen decision tree ‘Assessment of the Allergenic Potential of Foods Derived From Biotechnology’ (FAO/WHO 2001).

For exposure determination, the susceptibility of the AtNPR1 to proteolysis was tested as recommended by the EPA. This was tested in two ways: in silico and in an experimental digestion with proteases found in the human gut. Results from both tests confirm rapid digestion of AtNPR1 by the human digestive system. Given this rapid digestion, the exposure to AtNPR1 upon human consumption is likely very brief. The *NPTII* gene, encoding an aminoglycoside 3′‐phosphotransferase, is a widely used neomycin resistance selection marker and has been approved by the FDA as a food additive in GM cotton, oilseed rape, and tomato (U.S. Food and Drug Administration 2024). Altogether, these findings meet the criteria established by multiple lines of evidence required for transgenic crop safety assessments (Herman et al. 2006). Hence, from a consumer safety and regulatory perspective, our risk assessments support a determination that AtNPR1 does not pose a risk to human health or the environment.

Nutritional analysis of juice from transgenic citrus lines 35–30 and 24–25 was similar to the non‐transgenic 'Hamlin' control or USDA FDC ID:2003591 reference values for commercial orange juice (U.S. Department of Agriculture 2021). Overall, these results demonstrate that the *AtNPR1* transgene expression does not significantly alter the nutritional profile of the juice relative to standard commercial juice.

The genomic position of the T‐DNA insertion and orientation in each line was determined by whole‐genome sequencing and comparisons to the *C. sinensis* genome assembly reference DVS_A1.0 (RefSeq GCF_022201045.2). This was done primarily for the rapid identification of the lines in the field through PCR needed for regulatory or enforcement purposes. Two of the five lines, 26–36 and 35–30, studied here were found to be clones of each other. The T‐DNA insertion sites were identified as heterozygous and mapped to chromosomes 1 and 6, with no disruption of genes known to be directly linked to fruit development or yield. For genes with coding sequences that span both sides of the T‐DNA insertion – such as *RPS7* (40S ribosomal protein S7) and *HO1* (heme oxygenase 1, chloroplastic) – their function may be retained through redundancy (Degenhardt and Bonham‐Smith 2008; Gisk et al. 2009; Mulaudzi‐Masuku et al. 2019) or by the intact copy on the homologous chromosome.

One of the major challenges in commercialising GM crops is public perception and regulatory approval. While public opinion on GM crops in the U.S. is evolving, concerns regarding consumer acceptance persist (Kennedy and Thigpen 2020; Verma et al. 2021). The successful introduction of other GM fruits, such as the Arctic Apple (Stowe and Dhingra 2021), demonstrates a gradual shift toward acceptance, and this trend is expected to be even stronger when the modification addresses a critical agricultural problem like HLB. Given the severe economic impact of HLB on the citrus industry, the potential benefits of these transgenic trees are likely to outweigh potential risks. Nonetheless, fruit trees have long life cycles, and in the United States, multiple regulatory agencies, including the USDA, FDA, and EPA, must approve their environmental release, which can significantly delay market entry (Lobato‐Gómez et al. 2021).

In conclusion, this study provides strong evidence supporting the commercial viability of *AtNPR1* transgenic 'Hamlin' sweet orange. Ongoing and future research will further refine these findings and aid in the development of strategies to increase industry and consumer acceptance of this biotechnological solution for HLB management.

## 4 Methods

### 4.1 AtNPR1 and NPTII Protein Concentration in the Transgenic Citrus

One‐hundred mg of citrus leaf tissue and 300 mg of fruit pulp were ground with liquid nitrogen, and total protein was isolated using a methanol/acetone extraction at −20°C. Five mL of fresh juice was mixed with five volumes of pre‐chilled acetone at −20°C, and total protein was precipitated for 1 h at −20°C. The sample was centrifuged at maximum speed for 10 min at 4°C to pellet the protein. Pelleted total protein samples were dried using a rotary evaporator. Two hundred μL of protein Extraction Reagent Type 4 (Sigma Aldrich) was used to solubilise the pellet, and 20 μL of total extracted protein was denatured and loaded into SDS‐PAGE gels for Western Blot (WB). Protein concentrations were estimated using ImageJ (Rasband 2007) based on WB images containing both standards and samples. For the concentration calculations of AtNPR1, an initial 3 μg/μL of sfGFP‐NPR1‐His was serially diluted in 1:10 increments to generate standards and 30 μL of each was loaded in the gel. The lowest WB detectable band with a concentration of 9 × 10^−3^ μg/μL was normalised to 1.00. A ratio of the band intensity for each of the AtNPR1 samples from leaf, juice, and pulp tissues was generated and used to estimate protein concentration. NPTII protein concentration was estimated in a similar fashion.

### 4.2 AtNPR1 Protease Digestion Tests

Methods for digestion tests were recommended by the EPA and are described in the 'Pepsin Resistance' section of FAO/WHO (2001). Briefly, in vitro digestion of AtNPR1 by proteases was performed by treating 1.67 μg of sfGFP‐AtNPR1‐6His with either 0.085 μg of trypsin or pepsin (enzyme:protein = 1:20 w:w) for 0, 1, 2, 3 and 4 h, followed by analysis via WB.

### 4.3 AtNPR1 Allergen and Toxicology Testing

Allergen assessments of the AtNPR1 protein were performed using the web‐based tools AllergenOnline (Goodman et al. 2016) and Allermatch (Fiers et al. 2004), following standard guidelines from the FAO/WHO. The assessment searched for 80‐amino acid windows containing a minimum of 35% non‐continuous identity (Fiers et al. 2004). Toxicity assessments were conducted using CSM‐Toxin (Morozov et al. 2023) and ToxDL (Pan et al. 2021), with default settings. The AtNPR1 amino acid sequence from UniProtKB (accession P93002) was used as input where applicable. For ToxDL, structural data from the Protein Data Bank entry 7MK2, representing the AtNPR1 protein, was used.

### 4.4 Neighbour‐Joining Phylogenetic Tree of NPR1 Proteins and Nutrient Content of the Transgenic Lines

Protein alignment and phylogenetic tree were generated using Geneious Prime 2025.1.2. Pairwise distances between protein sequences were calculated using a BLOSUM62 cost matrix, and a Neighbour‐Joining tree was constructed. Seventeen of the 18 plant species represented in this phylogenetic tree are edible, including all the *Brassica* species and *A. thaliana* itself. A list of NPR1 protein sequence accession numbers and their identity and similarity percentages comparing each to *A. thaliana* NPR1 are presented in Table S1. Nutrient analysis of the transgenic fruit juice was conducted by the University of Florida Citrus Research and Education Center, Food Science and Human Nutrition Department. Nutrient content was determined using the Official Methods of Analysis (OMA) established by AOAC INTERNATIONAL (AOAC 2023).

### 4.5 Transgenic Line Production and Selection of HLB‐Tolerant Lines for Commercialisation

Previous work identified two independent *AtNPR1* transgenic lines of 'Hamlin'1 sweet orange exhibiting significant levels of HLB tolerance, which was retained to third‐generation progeny, as described by Robertson et al. (2018). These progeny lines (13–3 and 13–29) are still maintaining strong HLB tolerance and are expected to begin producing fruit in the Spring of 2025. Lines 24–25, 26–36 and 35–30 were produced using mature tissue transformation to expedite flowering and fruiting (Canton et al. 2024). All lines are currently in a field site at Picos Farms in Fort Pierce, FL, and those produced with mature tissue transformation are producing fruit. HLB symptoms are determined by examining the canopy (Slinski 2016). To determine *C*Las infection in the trees, real‐time qPCR was performed in triplicate according to the Thermo Scientific Maxima SYBR Green qPCR Master Mix (2×) protocols using a QuantStudio 3 with the *terC* primer set (Petrone et al. 2022).

### 4.6 T‐DNA Insertion Site Identification by Whole‐Genome Sequencing of the *At NPR1* Transgenic Citrus Lines

Two hundred mg of leaf tissue from each transgenic line was ground in liquid nitrogen, and genomic DNA was extracted using the DNeasy Plant Mini kit (Qiagen). For each line, three μg of DNA were sequenced using Oxford Nanopore Technologies (ONT) GridIon in two separate FLO‐MIN114 flow cells with SQK‐LSK114 chemistry, following manufacturer protocols. Basecalling was done in the MinKNOW v22.12.5, running Guppy v6.4.6 or higher with super‐accurate basecalling (260 bps). Reads in .fastq format were filtered to a minimum average Phred score of 15 using Filtlong (Wick 2025). Genome assemblies for each line were generated using the Flye assembler (Kolmogorov et al. 2019) with parameters: flye –nano‐hq –asm‐coverage 60 –genome‐size 370 m. The final assembly.fasta consensus file generated by Flye was used for subsequent analysis, as polishing with Medaka and/or Racon did not significantly improve assembly quality. Genome assembly quality was evaluated using Quast (Mikheenko et al. 2023), comparing each assembly against the *C. sinensis* genome assembly reference DVS_A1.0 (RefSeq GCF_022201045.2). Busco quality control scores were determined using the eudicots_odb10.2019‐11‐20 dataset. The entire target T‐DNA sequence containing the *AtNPR1* gene was mapped against each genome assembly file using Minimap2 (H. Li 2018), and SAMtools (Danecek et al. 2021) was used to generate a .fasta file containing the entire contig sequence carrying the T‐DNA sequence. Sequences flanking the T‐DNA insertion site were mapped to the *C. sinensis* reference genome mentioned above to determine the chromosome insertion site for the T‐DNA in each line. A repository with bash scripts used in this analysis is publicly available in GitHub (https://github.com/gluspaula/NuCitrus_scripts). Genes spanning and flanking the T‐DNA insertion site were identified using NCBI nucleotide BLAST, with the contig sequence flanking the T‐DNA as query. Several parameter combinations were explored, including searching without an organism restriction and filtering specifically for *C. sinensis* using all three ‘Program Selection’ modes. All gene hits were recorded. Finally, the predicted chromosome number and T‐DNA insertion site for each line were verified by visualising gene hits in the NCBI genome browser.

Gene expression of genes spanning the T‐DNA insertion sites was analyzed by qPCR. Total RNA was extracted from 30 mg of flash‐frozen leaf tissue, treated with DNase I, and quantified by Nanodrop. cDNA was synthesized from 1 μg RNA using the Luna Script RT SuperMix Kit (New England Biolabs), diluted fivefold, and used as template in triplicate reactions with SYBR Green Fast qPCR mix (ABclonal). FBOX served as an internal control, and reactions were run on a QuantStudio 3 system (Thermo Fisher Scientific).

### 4.7 PCR‐Based Markers to Genotype and Distinguish the *At NPR1* Transgenic Lines

Once the chromosome number likely to contain the *AtNPR1* gene was determined, Geneious Prime 2025.0.3 was used to visualise and further analyse this region. For each line, alignments of the contig, annotated T‐DNA sequence and chromosome were generated. Primer sequences were designed for a region spanning the chromosome and the T‐DNA right and left borders, and PCR was used to experimentally determine the insertion sites. For each final reaction, 1× Phusion Hot Start II DNA Polymerase, 0.5 μM of each primer and 10–50 ng of template DNA were used in a 50 μL final reaction volume. The thermocycling parameters were as follows: initial denaturation at 98°C for 30 s, followed by 30 cycles of 10 s denaturation at 98°C, 30 s annealing at 60°C and 40 s extension at 72°C, with a final extension at 72°C for 5 min. Sanger sequencing was performed by Eton Bioscience Inc. for each amplicon DNA obtained, and the longest, highest‐quality sequences were aligned back to the T‐DNA‐containing contig‐chromosome alignment. Primers whose amplicon sequences best spanned this alignment were selected as markers for the transgenic lines. Clonal identity of lines 26–36 and 35–30 was confirmed by aligning their contigs containing the T‐DNA to each other. Additionally, Sanger‐sequenced consensus amplicons from the RB and LB of lines 26–36 and 35–30 were aligned to the corresponding T‐DNA‐containing contig alignments for validation.

### 4.8 Accession Numbers

Line 13–3 PV789760; Line 24–25 PV789761; Line 26–36 PV789762; Line 35–30 PV789763; Line 13–29 PV789764.

## Conflicts of Interest

The authors declare no conflicts of interest.

## Supporting information


**Data S1:** pbi70394‐sup‐0001‐supinfo.docx.

## Data Availability

The data that support the findings of this study are openly available in GenBank at https://www.ncbi.nlm.nih.gov/genbank/, reference number PV789760, PV789761, PV789762, PV789763, PV789764.
