# Supplementary material for: Risk Evaluation and Molecular Characterisation of AtNPR1 Transgenic Citrus Lines Tolerant to Citrus Greening Disease
Source: Plant Biotechnol J. 2025 Oct 13;24(3):1223–33. doi: 10.1111/pbi.70394 (PMC12946480; doi:10.1111/pbi.70394)
Supplement: Supplementary file 1 — Data S1: pbi70394‐sup‐0001‐supinfo.docx. [file PBI-24-1223-s001.docx]

Supplementary materials

# List of Supplementary Tables and Figures

[Table S1. Pairwise protein identity and similarity percentages between NPR1 amino acid sequence of *Arabidopsis thaliana* and 17 other plant species. 2](#_Toc208241833)

[Table S2. Identification markers for each transgenic line. 4](#_Toc208241834)

[Figure S1. Allergenicity assessment of AtNPR1 protein using AllergenOnline. 6](#_Toc208241835)

[Figure S2. Allermatch allergenicity assessment of AtNPR1 protein. 8](#_Toc208241836)

[Figure S3. In silico toxicity screening of the AtNPR1 protein. 9](#_Toc208241837)

[Figure S4. Images show the canopy appearance and overall health of transgenic citrus lines 13-3, and 24-25. 11](#_Toc208241838)

[Figure S5. qPCR analysis of *Candidatus* Liberibacter asiaticus (*C*Las) titer in transgenic and control citrus trees in July 2024. 12](#_Toc208241839)

[Figure S6. Genes predicted at or near the T-DNA insertion sites in transgenic citrus lines. 13](#_Toc208241840)

[Figure S7. Gene expression analysis of genes interrupted by T-DNA insertions. 15](#_Toc208241841)

# Supplementary Tables

Table S1. Pairwise protein identity and similarity percentages between NPR1 amino acid sequence of *Arabidopsis thaliana* and 17 other plant species.

| Species | NPR1 Accession Number | Identity Percentage | Similarity Percentage |
| --- | --- | --- | --- |
| *Brassica juncea* | Q2HZ49 | 61.7 | 82.9 |
| *Arabidopsis thaliana* | P93002 | N/A | N/A |
| *Brassica campestris* | M4EFX7 | 71.7 | 86.5 |
| *Brassica oleracea* | A0A0D3E436 | 71.5 | 86.5 |
| *Capsicum annuum* | A0A2G2Z2M3 | 50.5 | 77.7 |
| *Carya illinoinensis* | A0A922E4K5 | 34.7 | 66.7 |
| *Citrus clementina* | V4VD78 | 27.5 | 53.6 |
| *Citrus sinensis* | A0A067DGX6 | 28.9 | 57.3 |
| *Glycine max* | B8XQT6 | 36.5 | 70.6 |
| *Lactuca sativa* | A0A9R1XAR0 | 36.9 | 67.7 |
| *Malus domestica* | A0A191XY03 | 47.6 | 75.8 |
| *Mercurialis annua* | LOC126679545 | 34.3 | 62.8 |
| *Phaseolus vulgaris* | V7BNH8 | 36.5 | 70.3 |
| *Pistacia vera* | LOC116114899 | 35.9 | 67.6 |
| *Quercus robur* | LOC126714203 | 51.7 | 78.4 |
| *Solanum lycopersicum* | K4CDU6 | 51.0 | 78.8 |
| *Solanum tuberosum* | A0A9F1UC91 | 51.3 | 78.7 |
| *Spinach oleracea* | A0A9R0IZH4 | 49.8 | 77.1 |

Table S2. Identification markers for each transgenic line.

| Line (border) | Primer set amplifying the chromosome and T-DNA at each border (5’ to 3’) | Amplicon predicted size (bp) | Sequenced amplicon Pairwise identity (%) |
| --- | --- | --- | --- |
| 13-3 (LB) | 5985F^†^: GATCGCCCTTCCCAACAGTT  9082R: CCCATAGATGTTGCCAATTCATGT | 1455 | 95.2 |
| 13-3 (RB) | 330F: TAGACCATTTGAACCGGGCC  834R^†^: TGCTCTTCGTCCAGATCATCC | ABSENT | ABSENT |
| 24 (LB) | 5985F  8235R: GGGGTGTGTATATATATCTATGTGTGT | 1071 | 99.4 |
| 24 (RB) | 617F: CGGGTTGAGTTAGGGGATCT  834R | 1246 | 97.0 |
| 26 (LB) | 5985F  9175R: ATGAATGTTGTGGCTTGCGC | 1847 | 99.5 |
| 26 (RB) | 784F: TGGAAGTTGCTCGCTTGTAGT  834R | 1234 | 97.7 |
| 35 (LB) | 5985F  8857R: ATGAATGTTGTGGCTTGCGC | 1847 | 97.1 |
| 35 (RB) | 469F: GCTGGTGGTCGAATGATTGG  834R | 1390 | 99.8 |
| 13-29 (LB) | 5985F  2977R: GGACTCGAACCCAAGCAACT | 647 | 97.2 |
| 13-29 (RB) | 785F: GGGGACGAGTGGTGTTCAAT  834R | 1065 | 96.5 |
| ^†^Primer sequences 834R and 5985F are located on the T-DNA sequence right border and left border respectively and are therefore the same primer sequence for all lines. | | | |

# Supplementary figures


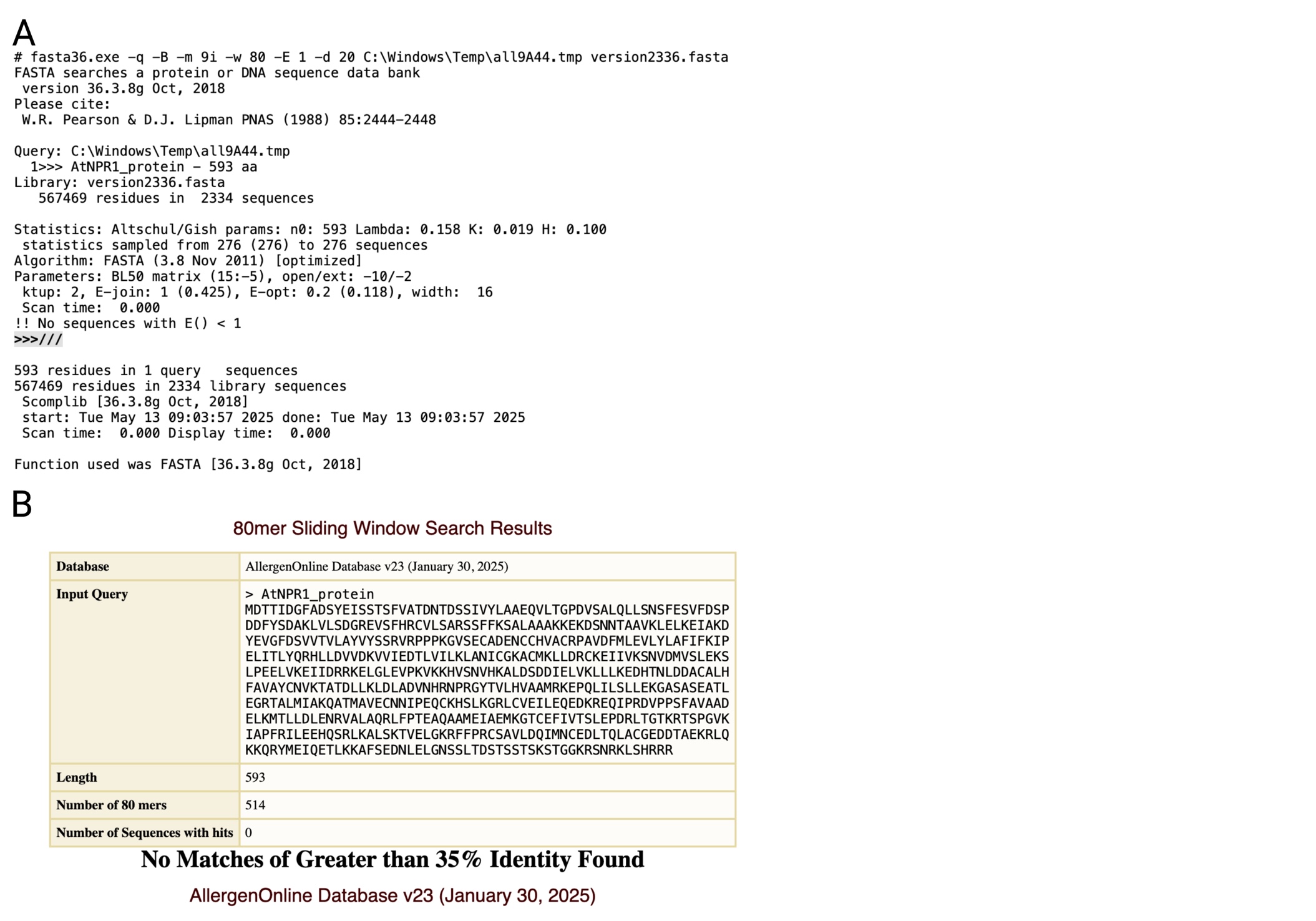


Figure S1. ****Allergenicity assessment of AtNPR1 protein using AllergenOnline.****

FASTA and 80-mer sliding window analyses were performed on the 593-amino-acid AtNPR1 protein using the AllergenOnline Database (v23, January 30, 2025). (A) No significant alignments (E-value < 1) were found in a FASTA search against 2,334 allergenic proteins, indicating no substantial homology. (B) An 80-mer sliding window comparison of 514 peptides revealed no matches with >35% identity, further supporting the absence of allergenic potential based on international safety thresholds.


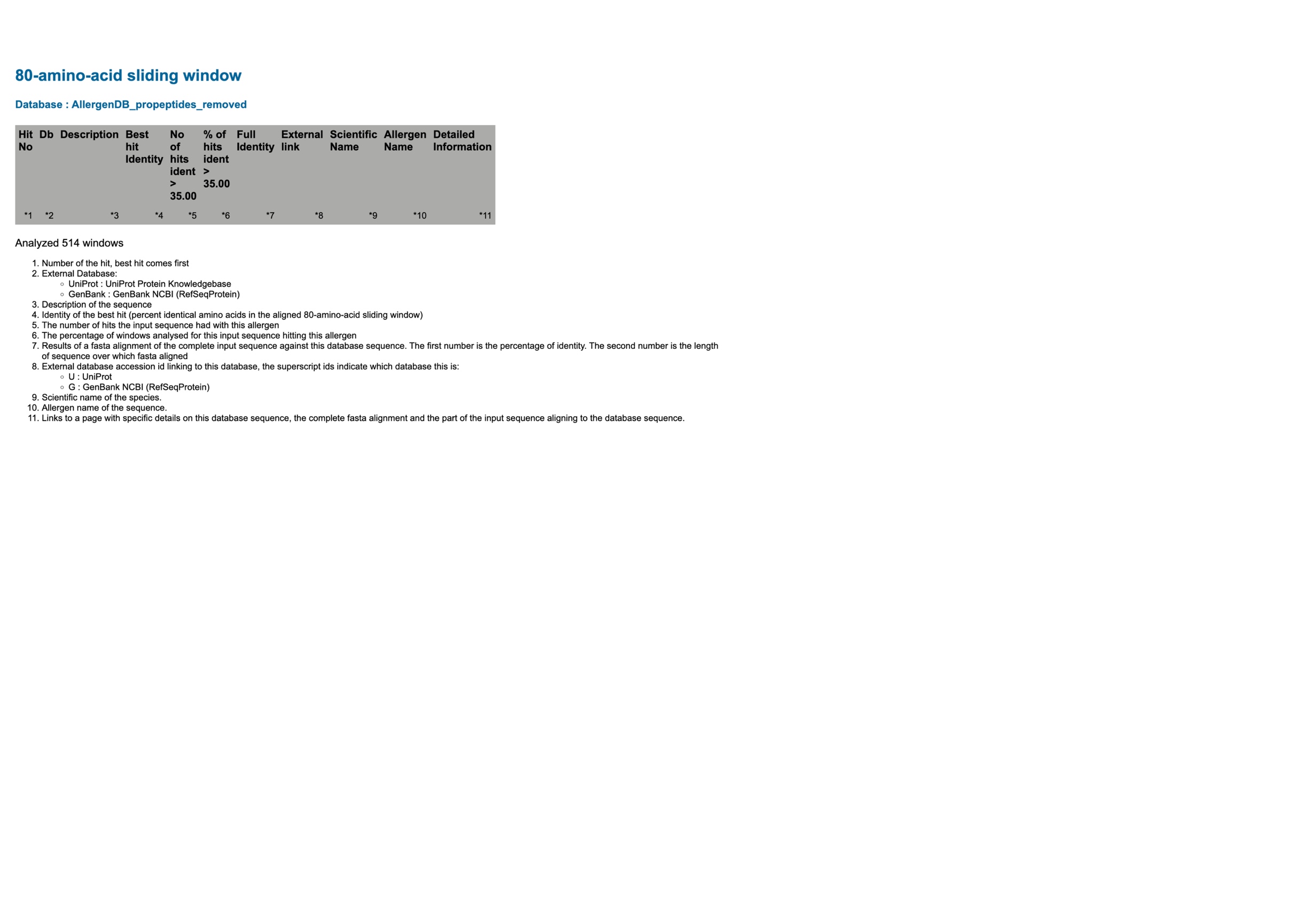


Figure S2. ****Allermatch allergenicity assessment of AtNPR1 protein.****

An 80-amino-acid sliding window analysis of the AtNPR1 protein was performed using the AllergenDB. A total of 514 windows were screened for similarity to known allergens. No matches exceeding the 35% identity threshold were found, indicating no significant allergenic potential based on sequence homology.


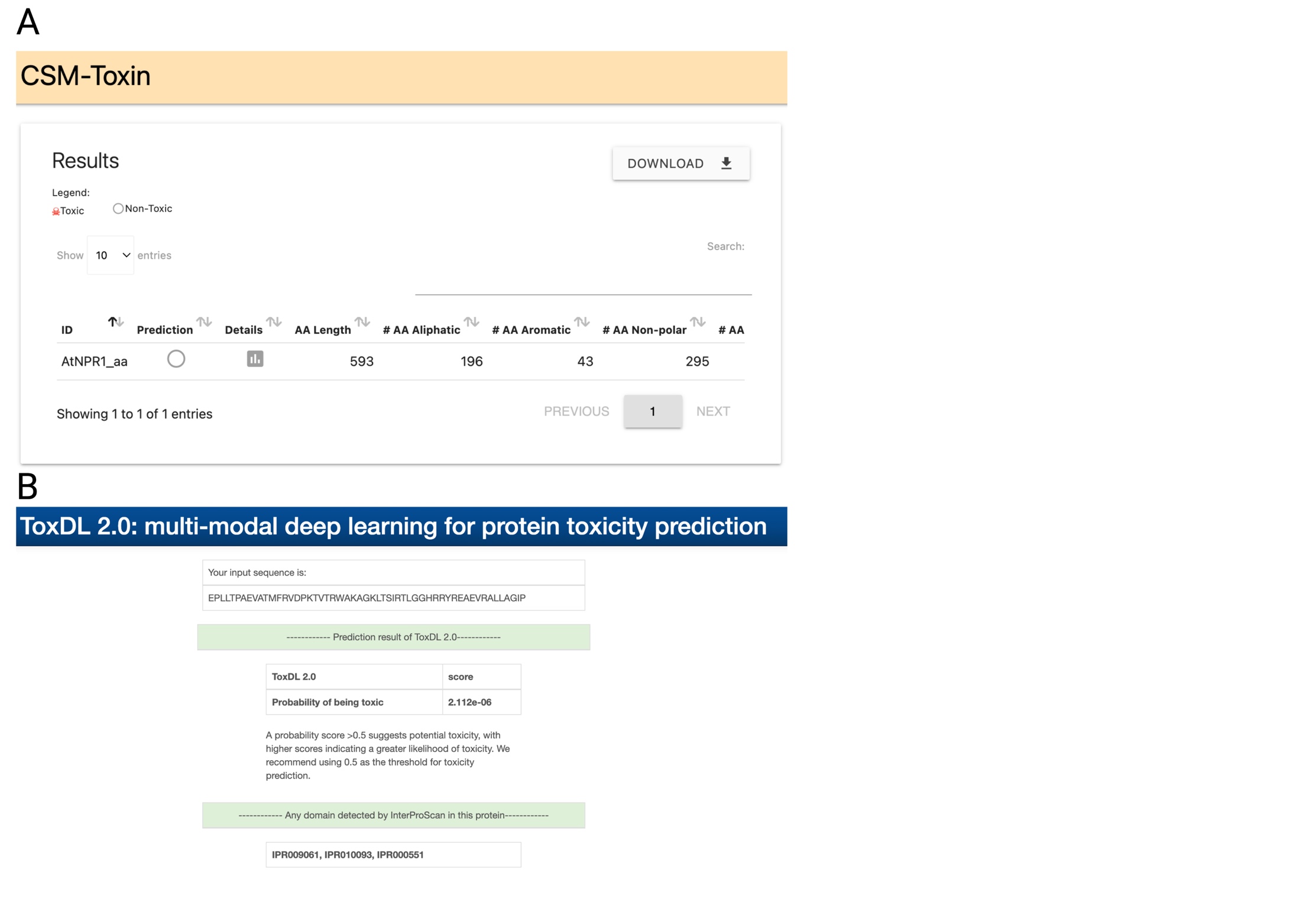


Figure S3. **In silico toxicity screening of the AtNPR1 protein.**

(A) CSM-Toxin prediction classified AtNPR1 as non-toxic based on amino acid composition and physicochemical features. (B) ToxDL 2.0 analysis, using structural information from the Protein Data Bank (PDB) entry 7MK2, yielded a probability score of 2.1×10⁻⁶—well below the 0.5 toxicity threshold—indicating no potential toxicity. InterProScan identified common protein domains with no toxic signatures.


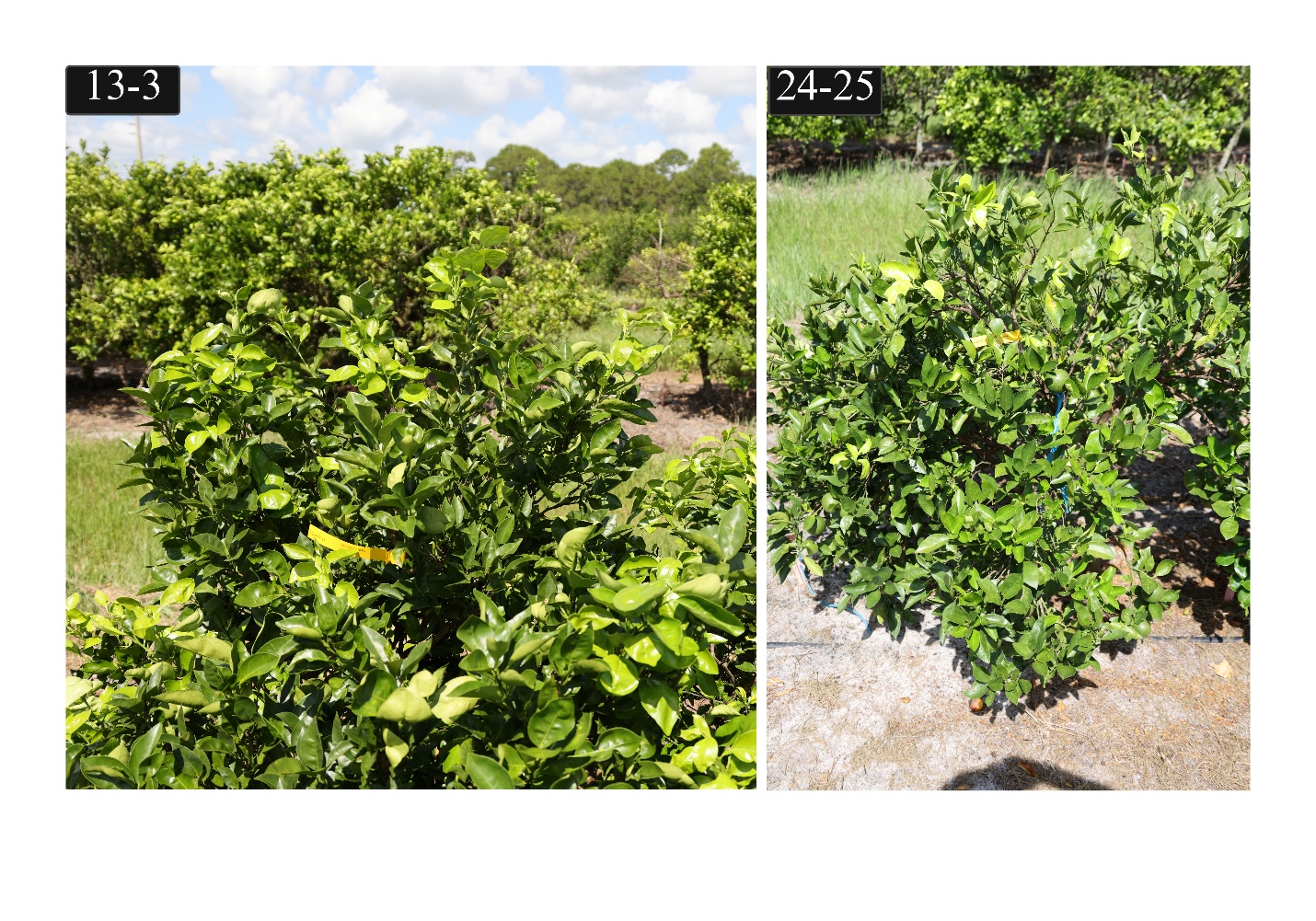


Figure S4. Images show the canopy appearance and overall health of transgenic citrus lines 13-3, and 24-25.

Images show the trees following multiple years of field exposure to natural infection pressure from *Candidatus* Liberibacter asiaticus (*C*Las). The trees exhibit full, green canopies with no visible symptoms of Huanglongbing (HLB), in contrast to symptomatic non-transgenic Hamlin control trees grown under identical conditions.


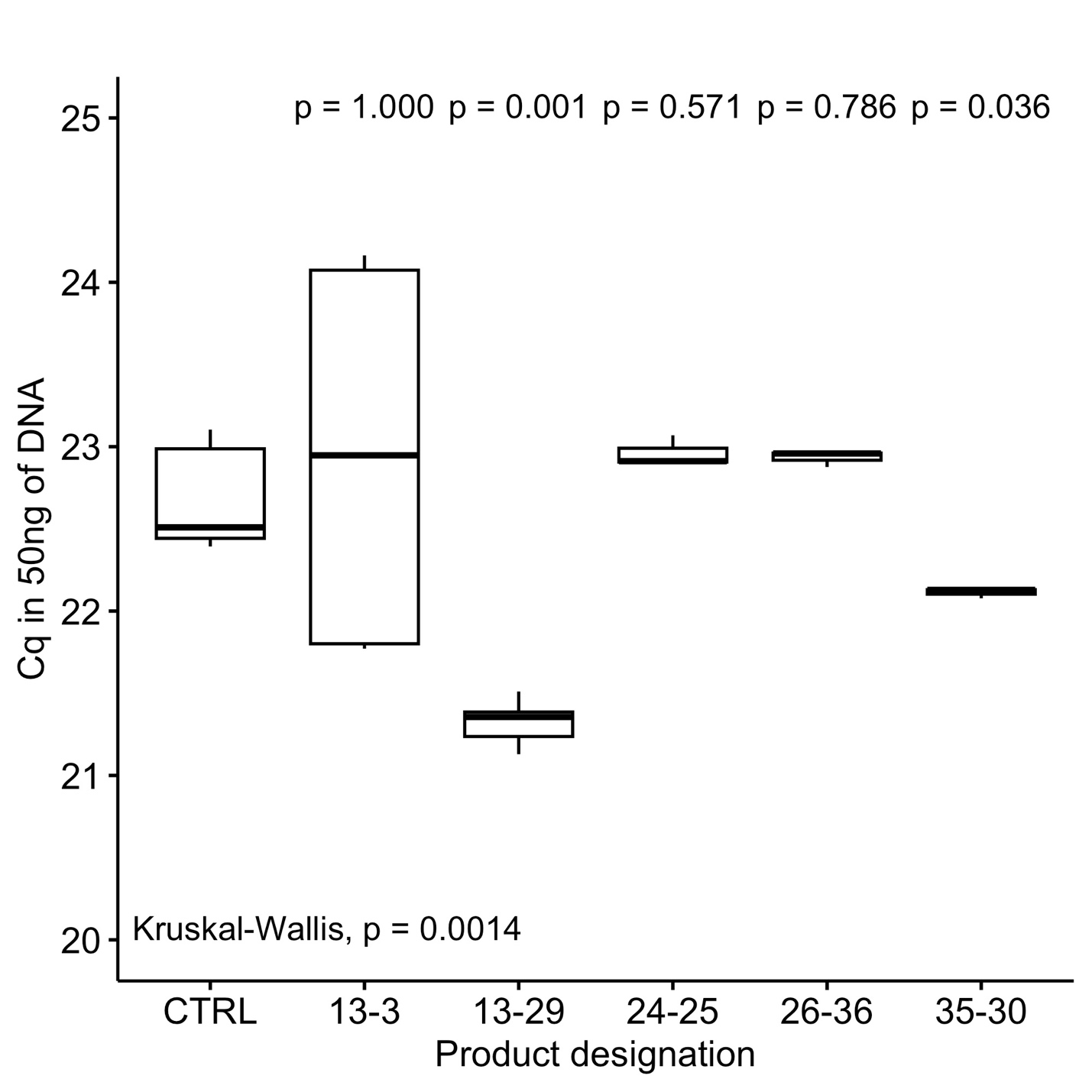


Figure S5. qPCR analysis of *Candidatus* Liberibacter asiaticus (*C*Las) titer in transgenic and control citrus trees in July 2024.

Boxplot shows quantification cycle (Cq) values obtained from qPCR targeting the *C*Las *terC* gene in 50 ng of total DNA from each citrus line and the non-transgenic control (CTRL). Higher Cq values indicate lower bacterial titer. The Kruskal-Wallis non-parametric group comparison (p = 0.0014) indicates significant overall differences among groups. Pairwise Wilcoxon test p-values comparing each transgenic line to the control are shown above the respective boxes.


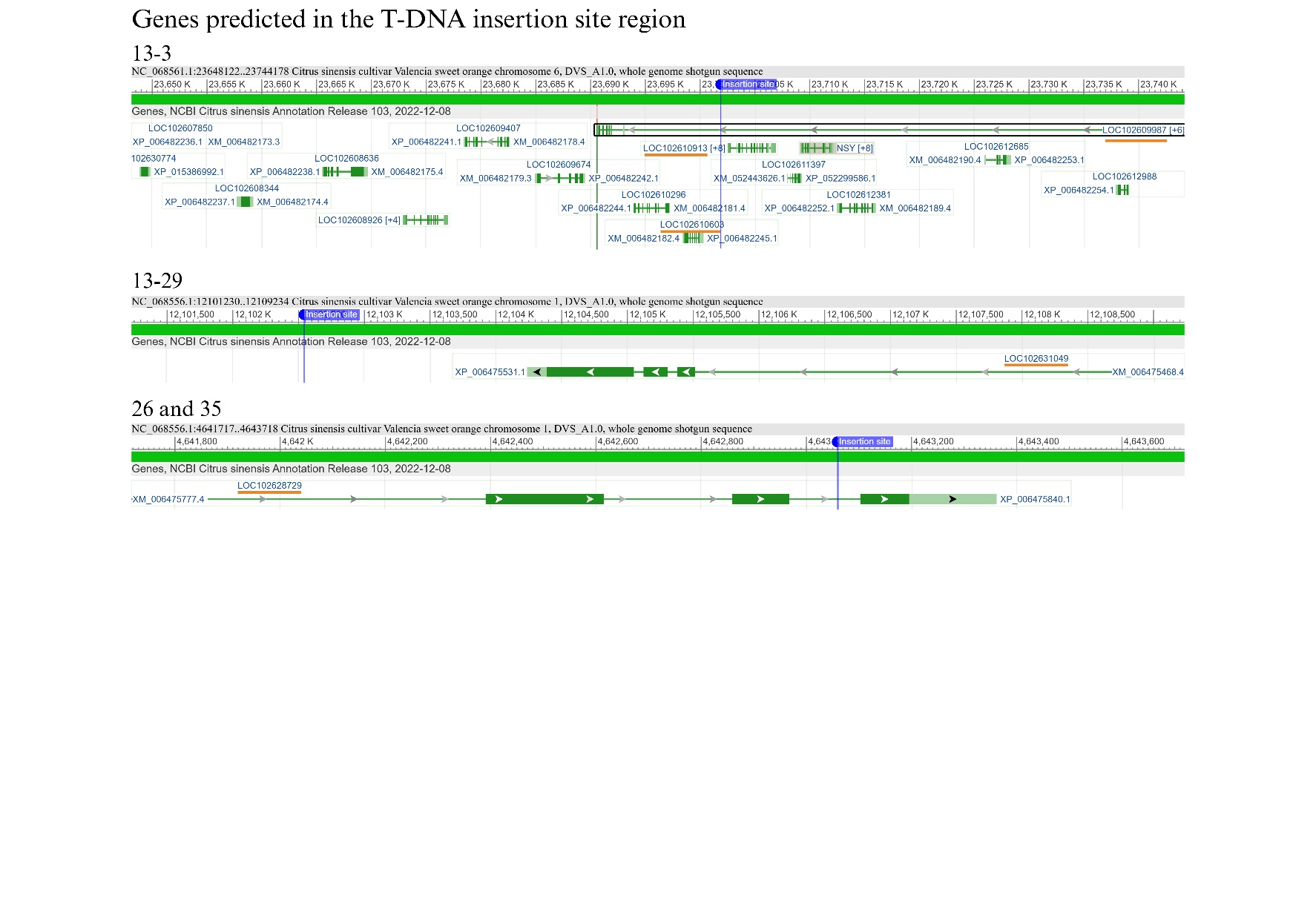


Figure S6. Genes predicted at or near the T-DNA insertion sites in transgenic citrus lines.

The *Citrus sinensis* reference genome DVS_A1.0 in the NCBI Genome Data Viewer was used to identify genes potentially affected by T-DNA insertion. The blue flag indicates the predicted T-DNA insertion site, and genes adjacent to or interrupted by the insertion are labeled with their corresponding LOC IDs (underlined in orange). For line 13-3 (top panel), the T-DNA right border (RB) is located on chromosome 6. Nearby genes include *Zinc transporter 5* and *Transcription factor bHLH75*. A *40S ribosomal protein S7* gene (*RPS7*) is located on both sides of the insertion site. In line 13-29 (middle panel), the insertion occurs on chromosome 1. The nearest gene, *12-oxophytodienoate reductase 3*, lies approximately 2 kb from the 5′ end of the insertion. For lines 26-36 and 35-30 (bottom panel), which were identified as identical insertional clones, the T-DNA integrates into the *heme oxygenase 1, chloroplastic* gene on chromosome 1. No genes were found at or adjacent to the insertion site in line 24-25 (not shown).


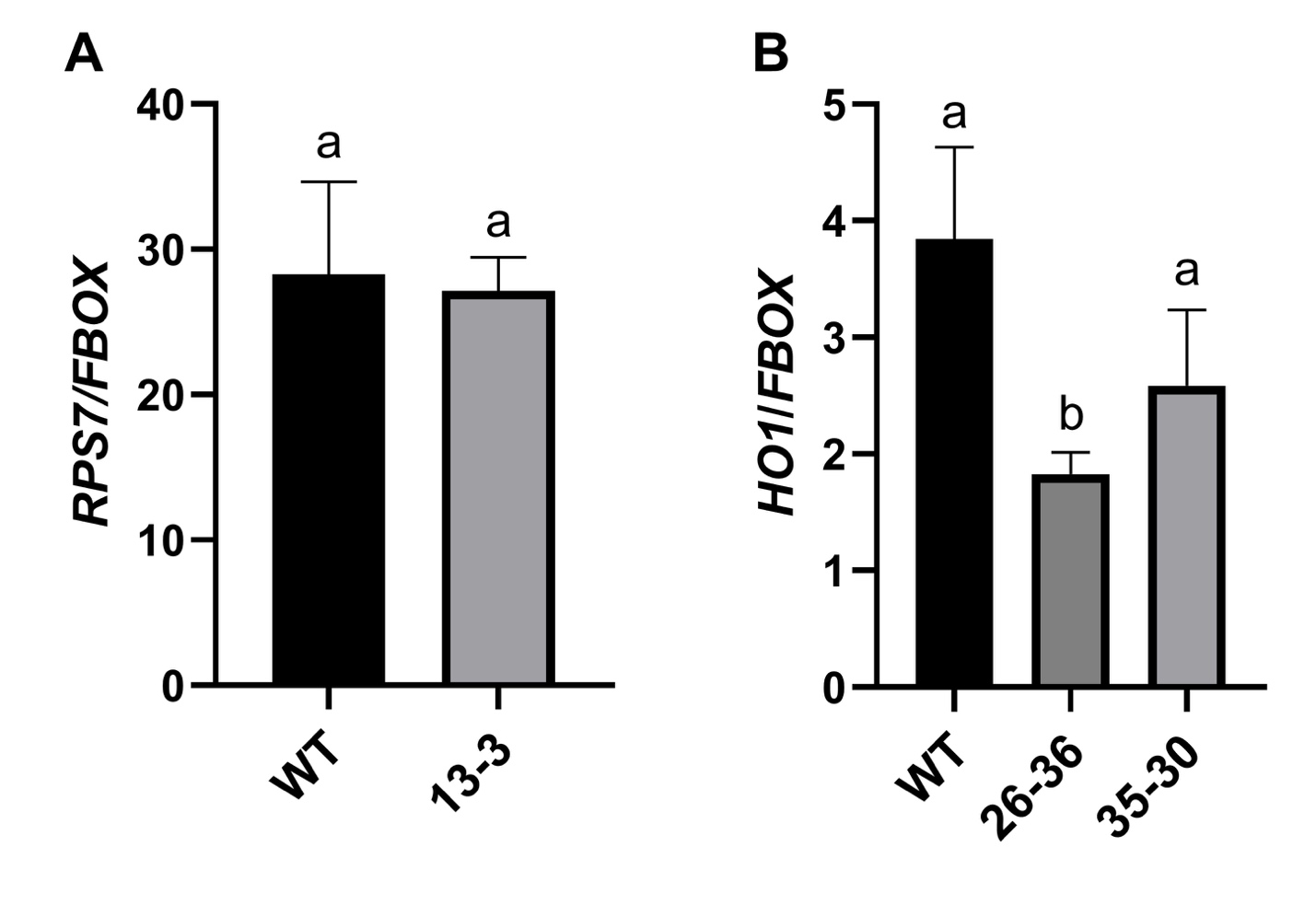


Figure S7. Gene expression analysis of genes interrupted by T-DNA insertions.

(A) The expression of *RPS7* in line 13-3 showed no significant difference from the wild type. (B) The expression of *HO1* in lines 26-36 and 35-30 was reduced relative to the wild type, though not significantly in line 35-30. Values represent means ± standard deviation of three biological replicates (Student’s t-test, *p* < 0.05).
